# Reelin and its receptors, VLDLR and ApoER2, in melanocytic nevi

**Published:** 2017

**Authors:** A Mihail, G Coman, F Staniceanu, L Coman, S Zurac, OA Coman

**Affiliations:** * Department of Dermatology, “Dr. Victor Babeş” Clinical Hospital of Infectious and Tropical Diseases, Bucharest, Romania; ** Department of Pathology, “Colentina” Clinical Hospital Bucharest, Romania; *** Department of Dermatology, “Titu Maiorescu” University, Bucharest, Romania; **** Department of Pharmacology, Faculty of Medicine, “Carol Davila” University of Medicine and Pharmacy, Bucharest, Romania; ***** Department of Physiology, Faculty of Medicine, “Carol Davila” University of Medicine and Pharmacy, Bucharest, Romania

**Keywords:** melanocytic nevi, reelin, VLDLR, ApoER2

## Abstract

Reelin is an extracellular signaling protein synthesized by Cajal-Retius cells in utero and early after birth, its presence being signaled in adult life too. Reelin acts on its receptors, VLDLR and ApoER2, acting on cytoskeleton, controlling migration and subsequently positioning and stabilizing the cortical neurons.

We investigated the reelin presence and its receptors, VLDLR and ApoER2, in melanocytic nevi considering the neural crest origin of the nevus cells and their migration into skin during embrionary period.

Melanocytic nevi present a strict cellular architecture and an increased malignant transforming capacity. We investigated reelin presence in 32 melanocytic nevi (5 junctional, 27 compound or 14 dysplastic nevi and 18 non dysplastic nevi). The assessment of reelin presence was performed by histological semiquantitative criteria.

Results showed the presence of reelin in 29 cases (29/ 32). The presence of reelin was elevated in junctional areas as in dysplastic nevi. VLDLR presented positive values in 16 cases (16/ 32) and ApoER2 was weak positive in 7 cases. Reelin or its receptors was peritumorally absent. Our study showed the presence of reelin in nevus cells from cutaneous melanocytic nevi and, in these cells, only the VLDLR receptor was present in half of the cases.

The significance of the reelin presence in cutaneous nevus cells may be hypothetically considered correlated with the position maintenance of the nevus cells or migration of these cells in malignant transforming situation.

**Abbreviations:** ApoER2 = apolipoprotein receptor 2, VLDLR = very low density lipoprotein receptor, DAB-1 = DIABLO protein, HMB45 = gene HMB45

Reelin is an extracellular signaling protein synthesized by the Cajal Retius cells in utero or early after birth, which controls the migration and the positioning of the cortical neurons [**1**]. Reelin acts by connecting to its receptors, VLDLR (very low-density lipoprotein receptor) and ApoER2 (apolipoprotein receptor 2), associated to the DIABLO protein (DAB-1). This results in the phosphorylation of DAB-1 tyrosine by the kinases of the src family [**2**]. The reelin-induced signal regulates the intracellular DAB-1 levels, with effect on the reorganization of the neuronal cytoskeleton, which is fundamental for the migration, positioning, and stability of the cortical neurons [**3**]. Reelin-deficient mutant mice show massive disorganization of the neurons from several cortical areas [**4**]. Beside the cortex, reelin can be temporarily seen in adult mice, in the peripheral nerves [**5**]. In adult mammals, reelin is also present in the liver; this organ may be considered a reelin source, the pituitary gland, and blood [**6**]. Reelin plasma levels (with alteration of reelin glicosylation) are increased both in experimental cirrhosis in mice and human cirrhotic patients [**7**]. 

Reelin was detected in the brain and CSF in adult humans [**8**]. 

Reelin was shown in several tumor cell types. Thus, epithelial prostate cancer cells show a massive expression of reelin. The reelin expression in these cells is significantly correlated with the Gleason score. Reelin was not expressed in benign prostatic proliferations [**9**]. Reelin is weakly expressed in gastric cancer, due to the aberrant hyper-methylation of the reelin-encoding gene promoter [**10**]. The increase in cellular motility and induced invasivity were obtained in pancreatic cancer by blocking ApoER2, VLDLR, and DAB-1 [**11**]. In breast cancer, the loss of reelin expression in tumor cells is associated with tumor proliferation. The increase in reelin expression in these cells is inversely correlated with the methylation of the reelin-encoding gene promoter. In vitro incubation of MDA-MD231 cells with recombinant reelin suppresses cellular migration [**12**]. 

Skin melanocytes result from neural crest by a long migration process during fetal development localizing at the skin area. Their epithelial localization is important because it forms a matrix of producing and distributing melanin to all keratinocytes. On the other hand, the positioning and maintaining of this position of naevic melanocytes onto basal membrane, as the eventual migration of these cells to superficial and profound dermis is important for tumoral proliferation of these cells. 

Little is still known regarding the presence and involvement of reelin in the maintenance of nevus architecture and in the proliferation, migration, and metastatic development of melanocytes from skin melanocytic nevi. These cells migrate into the skin during the embryonic stage, are strictly organized in cell nests, and are capable of malignant proliferation and metastatic development.

In our study, we investigated the reelin presence in melanocytes from cutaneous nevi considering that reelin may have an important role in malign or non-malign evolution of these naevi. 

## Patients and methods

We investigated 32 melanocytic nevi excised from 32 patients, in non-exposed body areas (13 men and 19 women aged between 16 and 54 years). Clinically, 18 patients showed regular melanocytic nevi, while 14 patients showed dysplastic nevi (minor to moderate architectural and cytological atypias). The classic histopathologic examination (paraffin sections stained with hematoxylin-eosin) confirmed the clinical examination: 14 dysplastic nevi (minor to moderate architectural and cytological atypias), 9 compound nevi, 5 junctional nevi, and 18 non-dysplastic nevi (17 compound and 1 junctional). Immunohistochemistry was carried out for S100, HMB45, melan A, Ki67, and c-Kit. Reelin and its receptors, VLDLR and Apo ER2, were determined by immunohistochemistry in all 32 nevi, both in the nevus melanocytes and in the peritumoral normal skin. Four μm thickness sections were treated with anti-reelin mouse antibodies (Santa Cruz Biotech., mouse citrate 1:500) and goat polyclonal IgG1 (Santa Cruz Biotech. 1:200). Staining intensity for reelin, VLDLR and ApoER2, was expressed according to semi quantitative criteria as it follows: negative, weakly positive (less than 25% positive cells noted “±”), average or moderately positive (26-50% positive cells noted “+”), high or intensely positive (over 50% positive cells, noted ” ++”). All the sections were independently assessed by two evaluators and negative controls were performed by substituting the primary antibodies with non-immune sera. All the patients signed the informed consent for biopsy investigation. The agreement for this study was obtained from the Ethical Committee of “Dr. V. Babes” Clinical Hospital. 

## Results

Reelin was found in 91% of the nevus cells (29/ 32) and was absent in 9% of the cases (3/ 32). The intensity of reelin expression in the nevus cells was intensely positive in 25% of the cases (8/ 32), moderately positive in 44% of the cases (14/ 32), and weakly positive in 22% of the cases (7/ 32) (**Table 1**). 

**Table 1:** Synthetic presentation of semiquantitative evaluation of immunohistochemical investigation

NEVI	CASES	AREA	REELIN				VLDLR				APOER2			
			++	+	+	±	++	+	+	±	++	+	+	±
JUNCTIONAL	6		5	1	0	0	0	0	6	0	0	0	3	3
COMPOUND	26	JUNCTIONAL	1	8	0	14								
		SUPERFFICIAL	2	5	4	4	0					0	4	0
		PROFUND	0	0	5	3		0	10	16	0			
DYSPLASIC	14		5	8	1	0	0	0	11	3	0	0	6	0
NON DYSPLASIC	18		3	3	9	3	0	0	5	13	0	0	1	

**Fig. 1:**
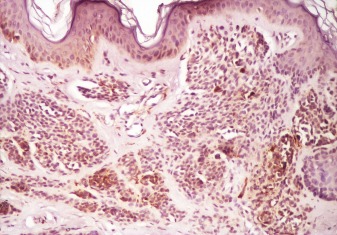
Reelin and its receptors: VLDLR and ApoER2 expression in melanocytic nevi a. Compound nevus with intense positive reelin at IHC junctional area with anti reelin antibodies (100X)

Reelin expression was absent in 9% of the cases (3/ 32). All the junctional nevi (6/ 32) and 23 of the compound nevi expressed reelin in the junctional area (9/ 32), in the superficial dermis in 15 cases (15/ 23) and in the deep dermis in 8 cases (8/ 23), respectively. In 4 cases, reelin was present both in the junctional area and the superficial area in the same compound nevus. The dysplastic compound and junctional nevi showed positive values for reelin in all cases: 37% (5/ 14) intensely positive, 57% (8/ 14) average, and 6% (1/ 14) weakly positive, respectively. Non-dysplastic nevi stained intensely positive for reelin in 16% of the cases (3/ 18), moderately positive in 16% of the cases (3/ 18), and weakly positive or negative in 50% of the cases (9/ 18 and 3/ 18, respectively) (**Fig. 1 a**,**b**,**c**).

Reelin was absent both in the dermis and epidermis in the peritumoral skin. VLDLR was shown in nevus melanocytes in 50% of the cases (16/ 32), with a weak staining intensity. All the junctional nevi stained weakly positive for VLDLR, as did 38% of the compound nevi (10/ 26). In dysplastic nevi, VLDLR stained weakly positive in 78% of the cases (11/ 14), and in 28% of the cases (5/ 18) of non-dysplastic nevi. Peritumorally, VLDLR stained weakly positive in 66% of the cases (8/ 12), while in 16% of the cases (5/ 32) VLDLR was weakly positive both within the tumor and peritumorally (**Fig. 1 d**,**e**). ApoER2 stained weakly positive in 22% of the cases (7/ 32), 3 of which were junctional nevi and 4 compound nevi, respectively, and 6 dysplastic nevi and 1 non-dysplastic nevus (**Fig. 1f**). ApoER2 was absent in the peritumoral skin. Biomarkers: S100, HMB45, and melanA were present in all the cases, while Ki67 showed values of less than 1% in 29 cases, 1-3% in 2 cases, and 12% in one case. The C-kit was significantly expressed in 17 cases (53%), 9 of which were dysplastic nevi and 8 non-dysplastic nevi. The intensely positive co-expression of reelin and c-kit was shown in 2 cases.

**Figure:**
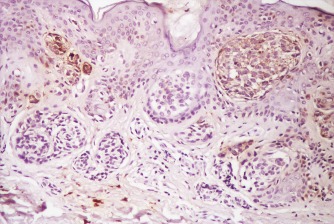
b. Compound nevus with intense positive reelin at IHC dermal area with anti reelin antibodies (100X)

**Figure:**
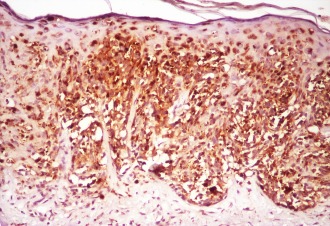
c. Junctional nevus with intense positive reelin at IHC junctional area with anti reelin antibodies (100X)

**Figure:**
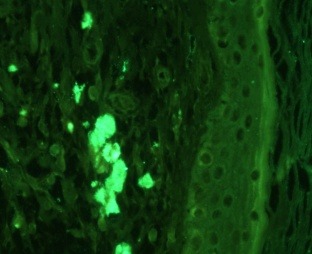
d. Compound nevus with rare VLDLR positive cells (weak positive). Immunofluorescence with anti VLDLR antibodies (200X)

**Figure:**
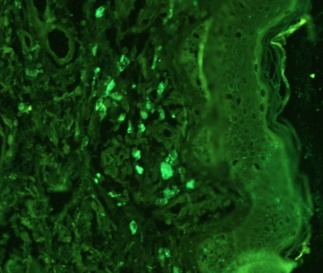
e. Compound nevus with rare VLDLR positive cells (weak positive). Immunofluorescence with anti VLDLR antibodies (200X)

**Figure:**
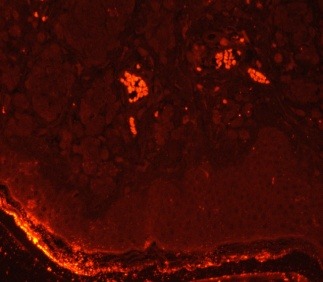
f. Compound nevus with rare ApoER2 positive cells (weak positive). Immunofluorescence with anti ApoER2 antibodies (200X)

## Discussions 

Our study showed that reelin was massively expressed in nevus cells (number of cases, intensity of expression). Topographically, reelin is particularly expressed in junctional nevi, as compared to the compound ones. Also, in compound nevi, reelin was mostly present in the junctional area versus the deeper dermal component, and showed similar placement with other melanocytic markers (e.g., HMB45). The VLDLR reelin receptor was similarly expressed as reelin, but in a much smaller number of cases and with a significantly lower intensity of expression. 

The presence of reelin in the nevus cells was correlated only with nevus dysplasia, assessed by clinical and histological criteria. The presence of reelin could not be correlated with the clinical appearance of nevi undergoing excision in non-dysplastic nevi. S100, HMB45, MelanA, Ki67, and c-Kit biomarkers showed no correlations with the presence or absence of reelin or its receptors.

The data collected in this study did not allow any assumptions regarding either the significance or the pathogenic role of reelin present in the nevus cells, or the origin of reelin in these cells. We may hypothetically presume that reelin has the same function in nevus cells as in the central nervous system, i.e., controlling the radial migration of neurons and their positioning during the embryonic stage, and maintaining the stability of neuronal positioning in adult life. 

In nevus cells, the coupling of reelin to its ApoER2 receptor results in the migration of nevus cells during the embryonic stage, while its coupling to the VLDLR receptor maintains the positioning of nevus cells in adult life. 

The increased expression of the VLDLR receptor vs. the ApoER2 receptor, seen in our study, supports this hypothesis. The opposing effects of the two reelin receptors are well known, since the coupling of reelin to its VLDLR receptor is followed by the phosphorylation of DAB1, resulting in migration arrest [**13**].

We may also hypothetically presume that, in certain normal or pathological circumstances of the adult life, reelin could promote cellular migration. Evidence supporting this hypothesis includes the presence of reelin in normal retinogenesis and its increased expression after retinal and corneal injuries. The authors suggested that reelin controls the migration of stem cells in neuronal and non-neuronal tissues during the adult life, in the same manner as during normal ontogeny [**14**].

On the other hand, the presence of reelin in internal organs of a non-nervous nature, including blood, suggests that the role of reelin could be a lot more complex.

An example is the presence of reelin as a matrix protein at collector blood vessels level. Reelin is involved in the cooperation between the endothelial cells and the smooth muscle cells without the implication of VLDLR and APOR2 receptors. Mice deficient in RLin gene present massive alterations of morphology and function of lymphatic vessels, meanwhile VLDLR and APOR2 deficient mice present a normal development of collector lymphatic vessels. On the other hand, reelin is involved in morphology and physiology of dermal collector lymphatic vessels and not at the nervous system level [**15**].

The association between reelin and cellular migration, both in the embryonic and adult life, led to the assumption that reelin could be involved in the proliferation and migration of tumor cells. Several studies associated the presence or absence of reelin in tumor cells with the malignant proliferation of these cells.

Thus, reelin was increased in prostate carcinoma, but not in benign prostatic hyperplasia. In gastric, pancreatic, and breast cancer, the decrease of reelin was associated with tumoral proliferation and increased invasivity.

Reelin was well expressed in our study involving nevocellular nevi – i.e., benign tumor with risk of malignant transformation. In one of our studies carried out in malignant melanoma (grade Clark IV invasive tumors), reelin was inconstantly expressed and unequally located within the tumoral mass. In some cases, reelin was absent or weakly expressed, but there were also other cases in which reelin stained moderately positive (unpublished data).

## Conclusions 

In conclusion, our data showed the significant presence of reelin and partially of its VLDLR receptor in the nevus cells, cells originating from the neural crest but situated outside the central nervous system. Reelin was not expressed in the peritumoral tissue. In the absence of more detailed data, it may be assumed that reelin could play the same role in nevus cells as it does in the central nervous system. 

In addition, reelin may be involved in maintaining and stability of nerves architecture by VLDLR receptor coupling and in some circumstances, it facilitates normal or tumor melanocyte migration. 

The demonstration of the presence of reelin in nevus cells, as well as the investigation of its role in neuronal migration in adult life, opened new study prospects in tumoral proliferation and invasivity.

The authors state no conflict of interest.
